# Comprehensive pathogen diagnostics in wild fish populations using blood-based molecular strategies: an Atlantic herring case study

**DOI:** 10.1038/s41598-025-28653-8

**Published:** 2025-12-31

**Authors:** France Caza, Fanny Fronton, Lina Ennia, Dominique Robert, Yves St-Pierre

**Affiliations:** 1 INRS-Center Armand-Frappier Santé Technologie, 531 Boul. des Prairies, Laval, Québec H7V 1B7 Canada; 2 Institut des Sciences de la Mer, Université du Québec à Rimouski, 310, allée des Ursulines, Rimouski, QC C.P. 3300, G5L 3A1 Canada

**Keywords:** Atlantic herring, Pathogens, *Ichthyophonus*, Erythrocytic necrosis virus, Transcriptomics, FTA cards, Diseases, Ecology, Ecology, Microbiology, Molecular biology

## Abstract

Climate change affects marine ecosystems by promoting pathogens that threaten key fish populations. To protect these, monitoring programs must adapt to manage threats and sustain fisheries. Here, we combined traditional PCR methods and transcriptomic analysis from a single drop of blood stored on FTA cards to determine the prevalence of erythrocytic necrosis virus (ENV) and the *Ichthyophonus* parasite in the Atlantic herring population. Across 2023–2024, 33% of individual blood samples tested positive for ENV and 10% for *Ichthyophonus* by PCR, with ENV-positive fish more frequently found in estuarine and coastal areas. Spatial analyses revealed a clustered distribution for ENV and a more sporadic occurrence of *Ichthyophonus*. RNA-Seq detected viral RNA fragments in ENV PCR-positive fish, revealing high levels of viral transcripts consistent with active viral replication. However, no significant changes were observed in the host blood transcriptome between infected and uninfected individuals, suggesting that ENV replication may proceed with limited systemic host transcriptional response under subclinical conditions. Overall, our study provides the first comprehensive baseline on the prevalence and molecular activity of ENV and *Ichthyophonus* in Atlantic herring, demonstrating the power of FTA-based RNA-Seq diagnostics to uncover hidden infections and informing future surveillance and management of wild fish populations.

**Supplementary Information:**

The online version contains supplementary material available at 10.1038/s41598-025-28653-8.

## Introduction

The impact of climate change on wild fish populations is becoming increasingly clear, and it has generally been described by shifts in species distribution, abundance, and food web dynamics^1,2^. Another threat to these populations due to climate change is the emergence of pathogens that endanger both adult and juvenile fish^3,4,5^. These effects have both economic and ecological consequences, as well as social impacts on communities that rely on commercial fishing.

Among pathogens affecting fish is the erythrocyte necrosis virus (ENV), a double-stranded DNA virus responsible for viral erythrocytic necrosis, which has been described as a temperature-dependent disease affecting Pacific herring^6^. The virus infects red blood cells, leading to a hemolytic disease that frequently causes anemia and increases their susceptibility to other stressors^7^. This virus is responsible for high mortality rates and plays a crucial role in the natural mortality of certain marine fish species. The fact that this virus is transmitted horizontally and is present in several fish species favors bidirectional transmission between species^8^.

While the prevalence of pathogenic viruses in finfish aquaculture, particularly in salmon, is well-documented, detecting these pathogens and assessing their impact on wild fish populations in situ faces significant challenges. These challenges stem from a combination of biological factors such as low viral loads and complex host-pathogen interactions, technological limitations in current diagnostic tools, logistical difficulties in sampling remote or diverse aquatic environments, and environmental constraints that affect viral transmission and persistence. Addressing these challenges requires a multifaceted approach that combines various diagnostic methods, each with specific applications, with a deeper understanding of viral ecology in aquatic ecosystems.

In the case of ENV, the traditional methods include microscopic examination of stained blood smears for characteristic inclusion bodies and electron microscopy to visualize the virions. PCR-based methods have also been developed for frozen tissues or tissues preserved using nucleic acid preservation methods to study the presence of this pathogen in herring populations on the Pacific coast^9,10^.

Another pathogen that has received attention is *Ichthyophonus hoferi*, a parasitic fungus-like organism that has caused significant morbidity in fish populations. Ichthyophoniasis has been reported as the cause of several episodes of mass mortality, with significant ecological and economic impacts, affecting Pacific herring and other species, including salmonids and crabs, along the West Coast of North America^5,11^. Like ENV, *I. hoferi* affects a wide range of marine and freshwater fish species, both in wild populations and aquaculture settings. The parasite exhibits different life stages, including a spore stage, which is particularly resistant to environmental stresses. Infected fish often exhibit a range of signs, including lethargy, abnormal swimming behavior, external cysts, and internal organ damage. The parasite primarily affects the fish’s heart muscle but can also infect other organs. Its detection involves a combination of clinical observation, histopathological examination, and PCR-based molecular techniques on tissue samples^12^.

Although the pathological, ecological, and economic consequences of the damage caused by these pathogens are relatively well understood, systematic monitoring programs, especially for wild fish populations, are generally lacking. This is partly due to the logistical requirements of such programs, which, in most cases, rely on tissue biopsies and the use of a cold chain during sampling, transportation, and storage.

In this study, we investigated the potential of employing a novel approach for detecting ENV and *Ichthyophonus* in Atlantic herring (*Clupea harengus*, Linneaus, 1758) from the Gulf of St. Lawrence, Canada. Atlantic herring, a pivotal species in marine food webs, plays a crucial role in the ecosystem structure and the economic stability of fisheries across the North Atlantic. However, like many marine species, they are susceptible to pathogens, which can influence the health status of individuals and population dynamics. Our approach, building on previous studies in fish^13,14^, employs a simple and cost-effective sampling strategy using a single drop of blood preserved on an FTA card. Combined with conventional PCR assays, this method enables the detection of *Erythrocytic Necrosis Virus* (ENV) and *Ichthyophonus* at the individual level and further allows comprehensive transcriptomic analyses that inform on ENV gene expression and replication, as well as on infection-associated changes in the host blood transcriptome. By integrating these advanced diagnostic tools, we aim to elucidate the spatial and temporal distribution of these pathogens and investigate their potential impacts on the host at the molecular level. We also took the opportunity to determine the biotic and abiotic factors associated with the presence of ENV and *Ichthyophonus* in herring from the Gulf of St. Lawrence.

## Results

### PCR detection of ENV and *Ichthyophonus*

**Fig. 2:**
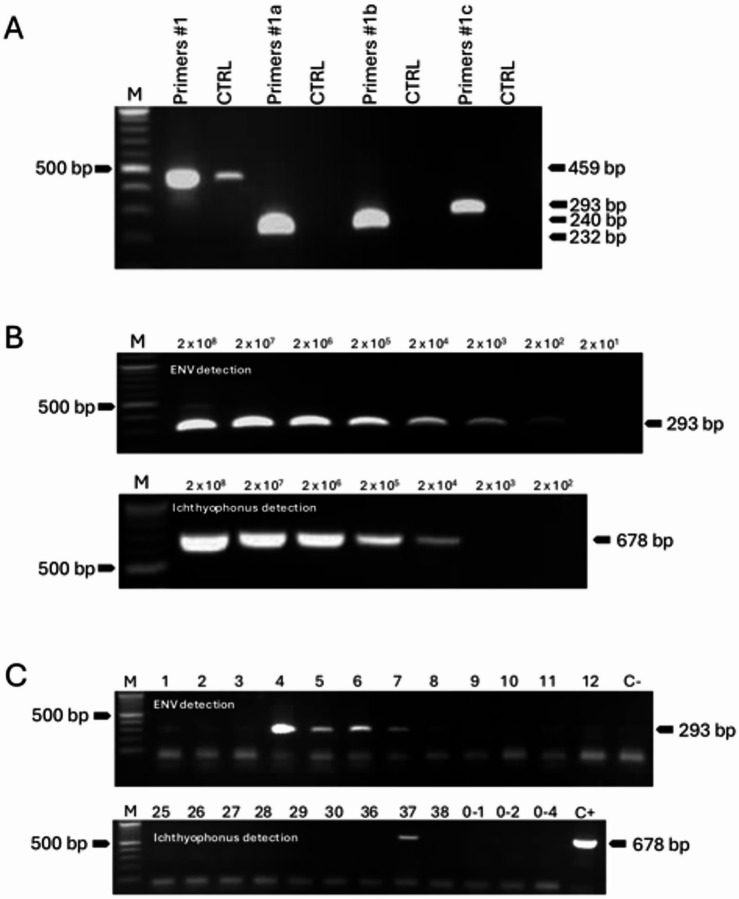
Development of ENV and *Ichthyophonus* detection tests by PCR. **(A)** PCR amplification using three sets of ENV-specific primers visualized on a 1.5% agarose gel. M indicates the molecular weight marker, and CTRL refers to the negative control. **(B)** Serial dilution test to assess the sensitivity of ENV and *Ichthyophonus* detection by PCR. **(C)** Screening of multiple samples for the presence of ENV (top gel) and *Ichthyophonus* (bottom gel). M: molecular weight marker. C- and C + indicate negative and positive controls, respectively.

Our first key questions were: How prevalent are ENV and *Ichthyophonus* pathogens among the two Atlantic herring populations of the Gulf of St. Lawrence, the spring and the fall spawners^15^? And, are positive cases randomly distributed or spatially structured? For the detection of ENV, specific PCR primers targeting a 293 bp region of the viral capsid protein were designed and chosen on criteria of the specificity to avoid cross-reaction with the host DNA and sensitivity (Fig. 2a). The sensitivity of the PCR assay, as determined by serial spiking dilutions of synthetic amplicons, was 2 × 10² copies (Fig. 2b). The same strategy was applied for *Ichthyophonus* detection, resulting in the identification of an optimal primer pair with a sensitivity of approximately 1 × 10² copies. Using these primers, 156 blood samples collected in 2023 were analyzed, yielding an overall prevalence of 48/156 (30.8%) ENV-positive and 18/156 (11.6%) *Ichthyophonus*-positive herring. Representative PCR results are shown in Fig. 2c. Amplicon identity using ENV- and *Ichthyophonus*-specific primers generating the 293 bp and 678 bp amplicons, respectively, was confirmed by Nanopore sequencing and subsequent BLASTn analysis (Supplementary Figures S1 and S2). Similar results (*p* = 0.23) were obtained with samples collected in the southern part of the Gulf during the 2024 survey, which showed a prevalence of 18/46 (39.1%) for ENV and 2/46 (4.3%) for *Ichthyophonus*-positive herring.

### Geographical distribution of ENV- and *Ichthyophonus*-positive herrings

**Fig. 3:**
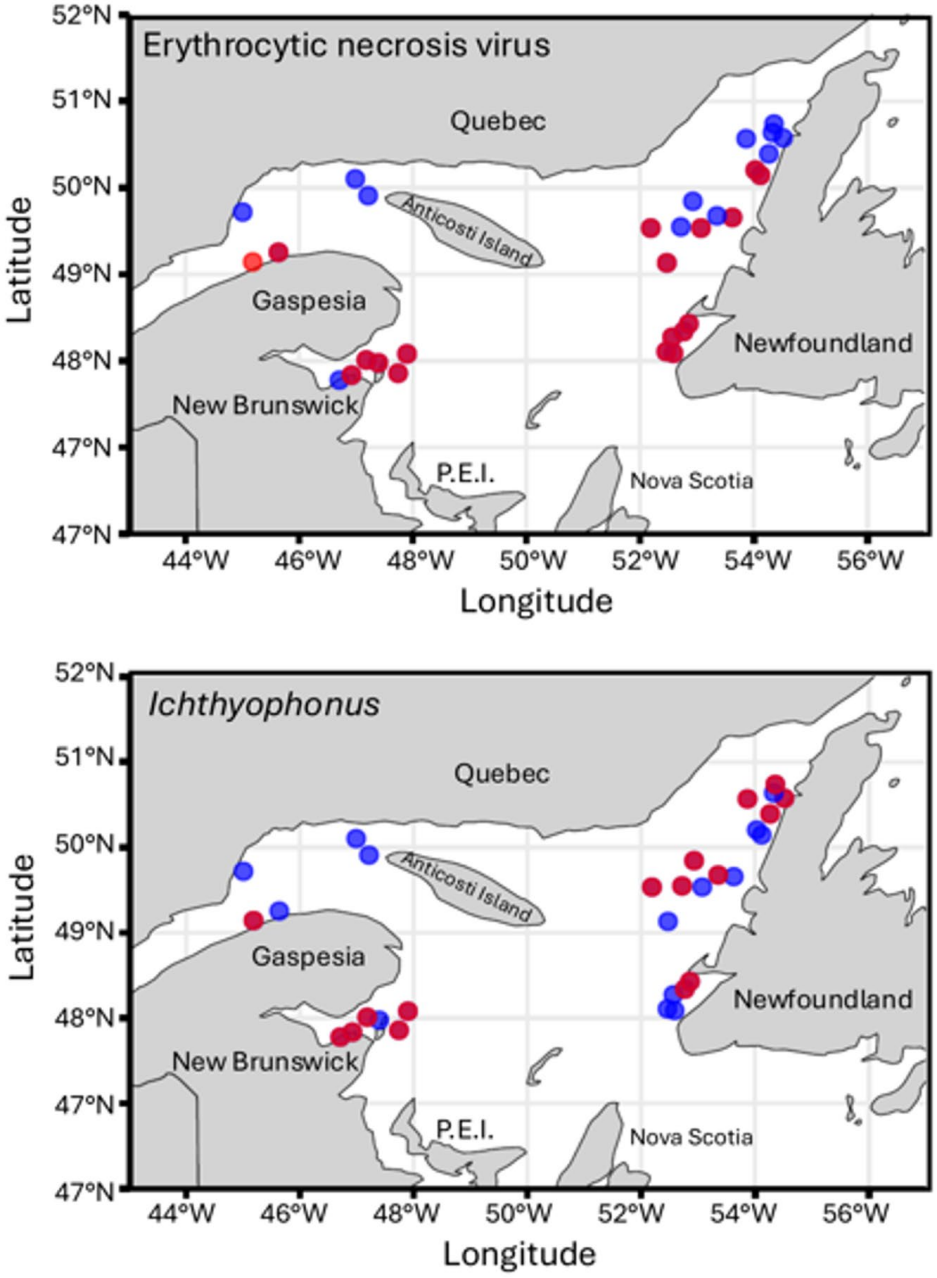
Distribution of herring sampling stations in the Gulf of St. Lawrence during the 2023 DFO survey. These maps show the distribution of ENV- and *Ichthyophonus*-positive (in red) samples in herring across various locations in the Gulf of St. Lawrence. Sampling focused on three main regions: the northwest part of the Gulf, north of the Gaspé Peninsula and near Anticosti Island; the northeast part of the Gulf, along the Newfoundland coast; and the south, in the Baie des Chaleurs, an arm of the Gulf of St. Lawrence situated between Gaspé (Quebec) and New Brunswick.

ENV positive samples were characterized by a widespread distribution, with clear clusters near estuarine and coastal regions (Fig. 3a). In the case of *Ichthyophonus*, a patchier distribution was observed, with clusters of high incidence interspersed with areas of low or no detection (Fig. 3b). Some sampling sites showed ENV positives without any corresponding *Ichthyophonus* positives. Spatial autocorrelation analysis using Moran’s I confirmed these observations: ENV showed significant clustering with a Moran’s I value of 0.24 and a highly significant p-value of 3.13 × 10⁻⁷, indicating strong spatial structure. Conversely, *Ichthyophonus* infections showed no significant spatial autocorrelation (Moran’s I = −0.086, *p* = 0.945), suggesting a random distribution. Taken together, these results indicate that ENV-positive samples are more spatially widespread and clustered, while *Ichthyophonus* positivity is relatively rare and more localized, likely reflecting distinct infection mechanisms.

### Biological and environmental factors affecting the prevalence of ENV and I*chthyophonus* in herring populations

**Fig. 4:**
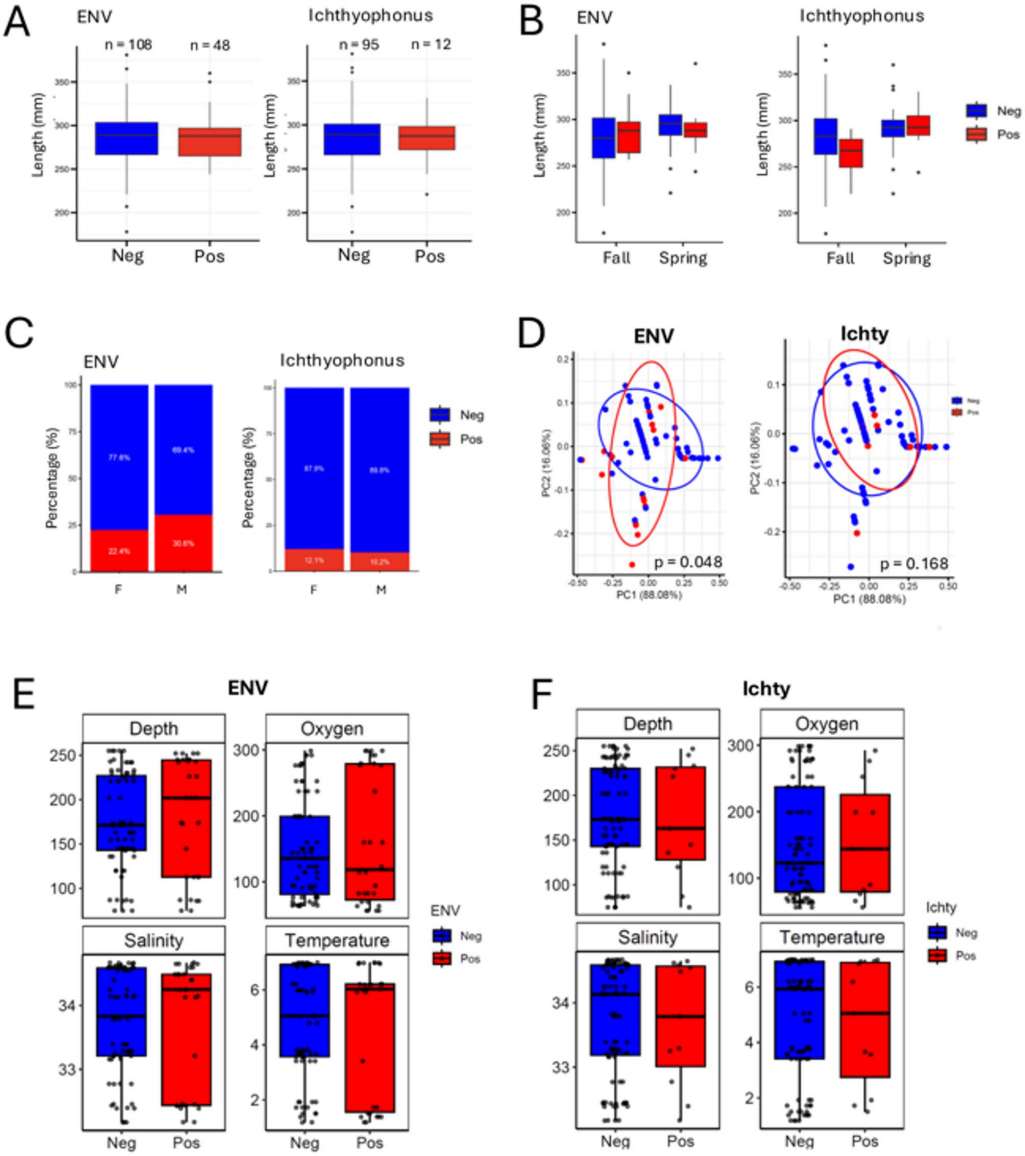
Associations between biotic and abiotic factors and infection rates of ENV and* Ichthyophonus *in Atlantic herring. **(A)** Comparison of the total length of ENV- and *Ichthyophonus*-positive samples, with sample sizes (n) shown for each group. The y-axis indicates fish length in millimetres. **(B)** Seasonal variation in the length of Atlantic herring negative and positive for ENV and *Ichthyophonus* during fall and spring. **(C)** Proportional representation of negative and positive cases of ENV and *Ichthyophonus* in female (F) and male (M) Atlantic herring. **(D)** Principal component analysis (PCoA) plots illustrating the clustering of negative and positive samples for ENV and *Ichthyophonus*. The percentage of variance explained by each principal component (PC1 and PC2) is shown, along with p-values indicating the statistical significance of the clustering. **(E and F)** Environmental conditions measured in Atlantic herring habitats, comparing fish negative and positive for ENV and *Ichthyophonus*, respectively.

We next examined whether the prevalence of ENV-positive and *Ichthyophonus*-positive fish varied with specific biological traits, such as length and gender distribution, and environmental variables. For these analyses, we used the samples collected during the 2023 campaign (*n* = 156). We found that the proportion of fish testing positive for either ENV or *Ichthyophonus* was not related to length (ENV: *p* = 0.92, *Ichthyophonus*: *p* = 0.40) (Fig. 4a**)**. Further comparison between the Spring and Fall spawning groups, which correspond to genetically and ecologically distinct stocks in the Gulf of St. Lawrence^15^, also revealed no significant difference in ENV or *Ichthyophonus* prevalence (Fig. 4b). Furthermore, the data indicated that the sex distribution within these spawning periods had no significant effect on the prevalence of ENV or *Ichthyophonus* infections (ENV: *p* = 0.9564, *Ichthyophonus*: *p* = 0.402) (Fig. 4c). Principal Coordinates Analysis (PCoA) based on Gower distances indicated a slight but statistically significant clustering of ENV-positive individuals (*p* = 0.048) (Fig. 4d), suggesting a minor structuring effect of ENV infection within the biological context of the samples. In contrast, no significant structuring was observed for *Ichthyophonus* (*p* = 0.168), indicating its more random and biologically neutral presence within these populations. Environmental variables, including depth, temperature, salinity, and oxygen concentration, also showed no significant differences in the percentages of ENV-positive or *Ichthyophonus*-positive fish (ENV: all *p* > 0.4, *Ichthyophonus*: all *p* > 0.8) (Fig. 4e **and f**). These findings suggest that the prevalence rates of ENV and *Ichthyophonus* infections are not significantly correlated with the examined abiotic factors or biological traits in the sampled population. This lack of correlation may partly reflect the highly migratory nature of herring within its distribution area.

### Influence of ENV and *Ichthyophonus* infections on herring blood microbiome

**Fig. 5:**
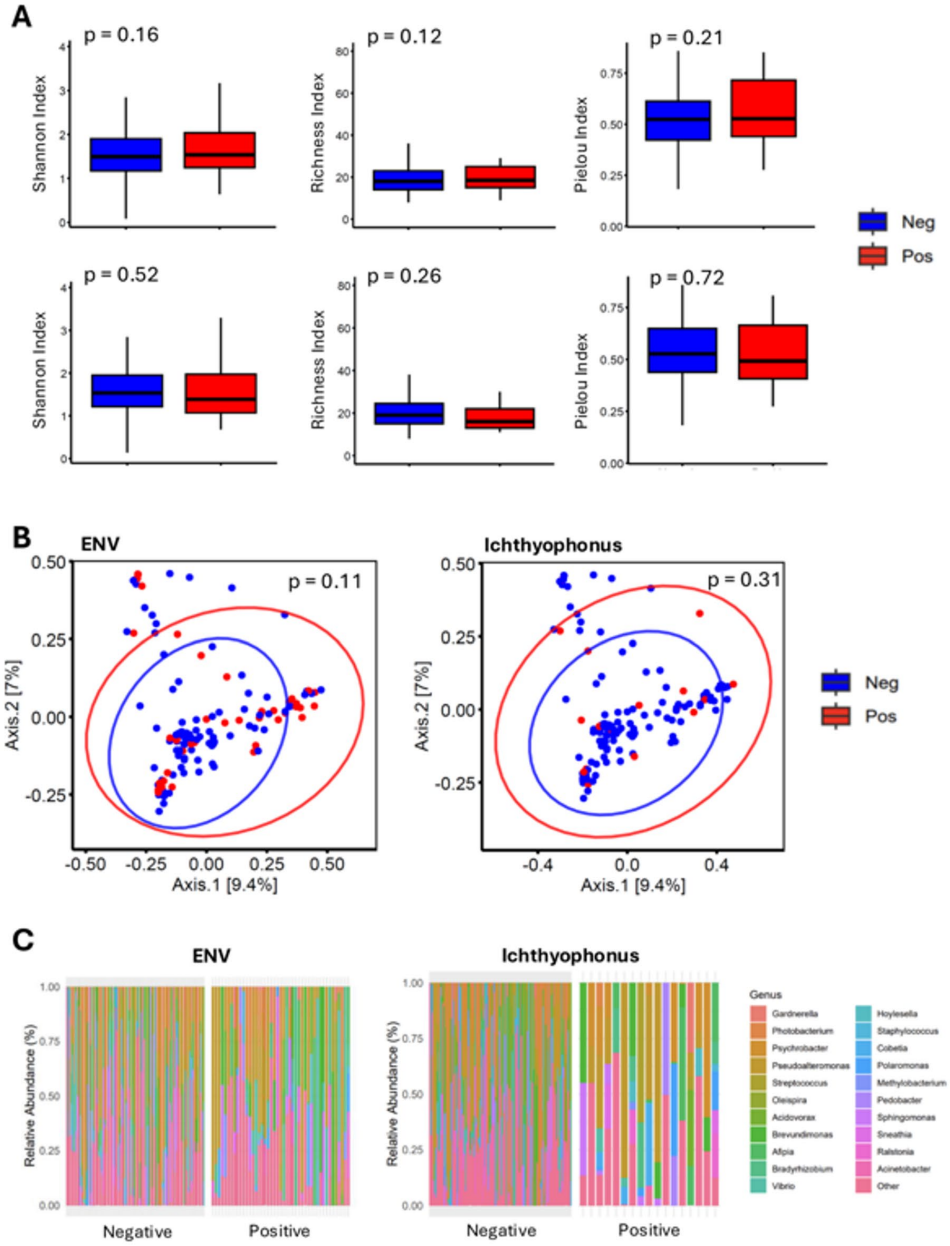
Comparative analysis of microbial diversity and community structure in Atlantic herring infected with ENV and I*chthyophonus*. **(A)** Microbial diversity. The top row shows the Shannon index, Richness index, and Pielou evenness for ENV-positive fish, while the bottom row shows the same indices for *Ichthyophonus*-positive fish. **(B)** PCoA plots showing the beta diversity of microbial communities associated with Atlantic herring. The left plot depicts communities from fish tested for ENV, and the right plot shows communities from fish tested for *Ichthyophonus*. Each point represents an individual fish’s microbial community. Ellipses indicate the 95% confidence interval around the centroid of points for each group. P-values evaluate the significance of community differences between negative and positive groups. **C)** Relative abundance of microbial genera in the blood microbiome of Atlantic herring, amplified using V3-V4 regions of 16 S rRNA.

The microbiome plays a significant role in the host’s immune system and overall health. Alterations in the microbiome can influence disease susceptibility, immune response, and pathogen dynamics within the host. Because ENV is a viral disease known to affect the erythrocytes of fish and that *Ichthyophonus* systematically infects various organs, we examined possible dysbiosis in ENV- and *Ichthyophonus*-positive fish. For this, we examined the blood microbiome. Recent studies suggest that the circulating microbiome primarily represents a transient and intermittent transfer of bacteria (or their genetic derivatives) across different tissue compartments^16^. A total of 5 fish out of 136 were found positive for both ENV and *Ichthyophonus* infection. To ensure the statistical independence of the two pathogen group comparisons (ENV status vs. *Ichthyophonus* status), these five co-infected individuals were excluded from all comparative microbial diversity and taxonomic composition analyses. We first examined alpha diversity and found no significant difference in microbial diversity between ENV-positive and ENV-negative individuals (Fig. 5a), or between *Ichthyophonus*-positive and -negative individuals. Beta diversity was evaluated using Bray-Curtis distances and Principal Coordinates Analysis (PCoA). For ENV status, a slight separation was observed, but with substantial overlap (Fig. 5b). The PERMANOVA test confirmed the absence of statistical differences (*p* = 0.095–0.105; R² = 0.0095). Similar results were obtained for *Ichthyophonus* (*p* = 0.555, R² = 0.004). The taxonomic composition at the genus level revealed no statistically significant differences in the microbial profiles between the positive and negative groups for both pathogens (Fig. 5c). Furthermore, under the stringent inclusion criterion of 0.01% relative abundance in more than 50% of all samples, no core blood microbiome could be identified across the cohort or within any subgroup—unlike observations reported in halibut from the St. Lawrence^17^. The absence of a consistent core community, together with the marked inter-individual variability, precluded the reliable identification of a dominant genus without introducing statistical bias. These results suggest that the circulating blood microbiota in Atlantic herring is largely composed of transient, low-abundance taxa rather than a stable microbial consortium.

### Differential analysis of ENV-positive vs. negative group transcriptome

**Fig. 6:**
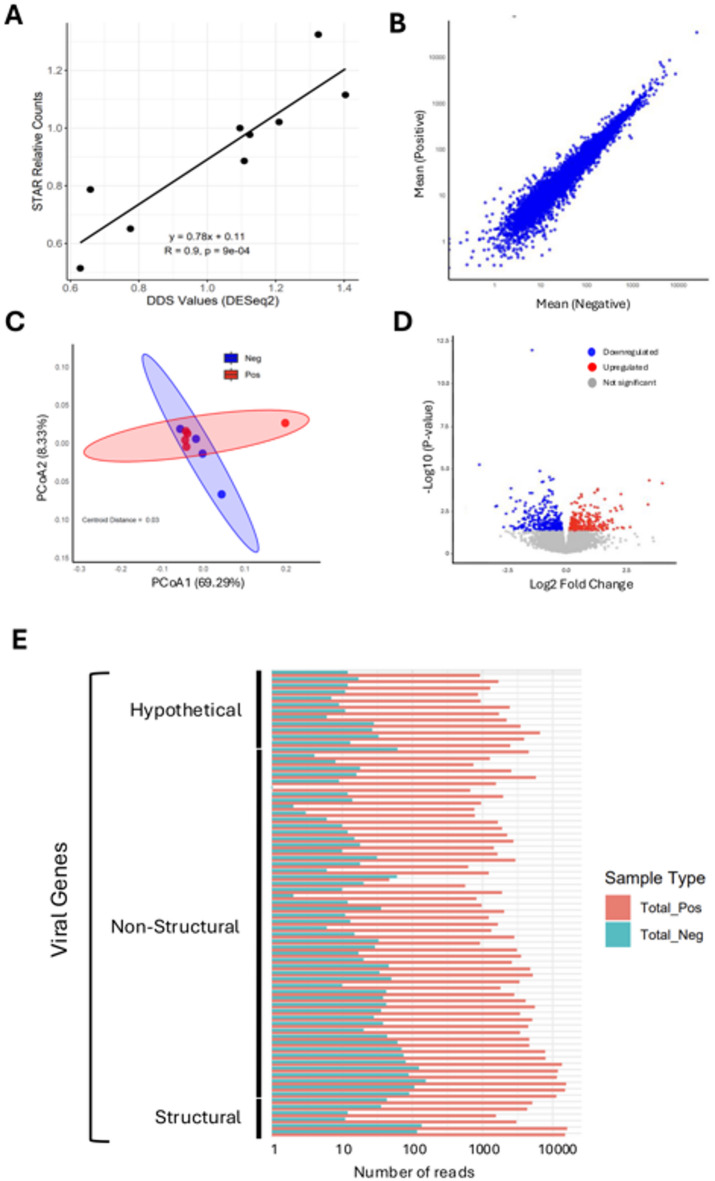
Transcriptomic profiling and viral gene detection in fish blood cells. **(A)** Scatter plot showing the correlation between standardized total RNA counts (STR) and differential expression scores (DSS values from DESeq2) across samples. The line represents the regression fit, with the coefficient of determination (R²) and p-value indicating the strength and significance of the correlation. **(B)** Relationship between the mean expression levels in negative samples (x-axis) and the log ratio of expression differences between ENV-positive and negative samples (y-axis). Each dot represents a gene. **(C)** PCoA plot showing the overall transcriptomic differences between ENV-negative and ENV-positive samples. Each point represents an individual sample, with ellipses showing the 95% confidence areas around group centroids. **(D)** Volcano plot representing the statistical significance (-log10 p-value) against the magnitude of expression change (log2 fold change) for each gene. Blue dots indicate downregulated genes, red dots show upregulated genes, and grey dots represent genes with no significant changes. **(E)** Bar chart comparing the number of reads obtained by RNA-Seq analysis in PCR-positive and negative samples, categorized into structural, non-structural, and hypothetical gene categories.

Given that the ENV virus produces systemic infection, we investigated whether the presence of ENV would influence the transcriptome of blood cells. For this purpose, total RNA was extracted from blood of ENV-positive and ENV-negative herring (excluding *Ichthyophonus*-positive individuals) and analyzed by RNA-Seq. A correlation plot showed strong agreement between DESeq2-normalized counts and STAR-assigned read counts (R² = 0.67, *p* < 0.01) (Fig. 6a). Scatterplots of gene expression revealed highly similar overall profiles between ENV-positive and negative groups (Fig. 6b). Although some genes exhibited low raw p-values and notable fold changes, none remained significant after FDR correction. PCoA analysis of transcriptomic profiles showed no significant separation between groups (PERMANOVA *p* = 0.98, R² = 0.056) (Fig. 6c), and a volcano plot confirmed the absence of strong differentially expressed genes (Fig. 6d).

We took this opportunity to determine whether we could detect viral RNA derived from the virus’s double-stranded DNA genome. Our BLASTn analysis of transcriptome reads against known ENV sequences revealed a strong enrichment of ENV-related hits in samples classified as ENV-positive. Positive samples exhibited between hundreds and several thousand ENV-aligned reads per file, with mean values ranging from approximately 432 to 1842 reads (Fig. 6e), providing evidence of active viral replication. Notably, ENV sequences were also detected at very low levels (10^2^ to 10^3^ times lower) in PCR-negative samples. Functional classification revealed that the majority encoded hypothetical proteins and proteins involved in virus replication.

## Discussion

This study on detecting viral and parasitic pathogens in Atlantic herring led to several significant findings. Firstly, we found that ENV and *Ichthyophonus* are prevalent among Atlantic herring in the Gulf of St. Lawrence. Our results suggest that up to 30.8% of Atlantic herring individuals test positive for ENV, while 11.6% test positive for* Ichthyophonus* among the sampled herring. This indicates that these pathogens are relatively common in Atlantic herring. Secondly, for ENV, we found a widespread and clustered distribution, particularly near estuarine and coastal regions. This pattern likely reflects specific environmental or ecological factors favorable to the transmission or survival of the virus, distinguishing it from the more randomly distributed *Ichthyophonus* infections. Thirdly, our RNASeq analysis revealed that ENV-positive fish showed elevated levels of viral transcripts, consistent with active viral replication. Our findings provide valuable insights into the epidemiology of ENV and *Ichthyophonus* in Atlantic herring, expanding the known geographic distribution of these pathogens in herring across North America.

### The case of ENV

The presence of the ENV virus has been well studied in Pacific salmon and Pacific herring (Pagowski et al., 2019), but very little research has been conducted on its occurrence in Atlantic species. As a generalist pathogen, ENV affects over 20 species of marine and anadromous fishes in the North Atlantic and North Pacific Oceans. Among the species encountered in the St. Lawrence Gulf, Pollock (*Pollachius virens*), Sandlance (*Ammodytes* spp.), Stickleback (*Gasterosteus aculeatus*), Atlantic cod (*Gadus morhua*), and Atlantic Salmon (*Salmo salar*) have all been previously described as infected by ENV^18,19^. Furthermore, Isopods of the genus *Gnathia* are known to act as vectors and can transmit ENV^20^. A comprehensive study of 14 infectious agents in Atlantic salmon (*Salmo salar*), spanning from Greenland to New Brunswick, did not detect ENV^21^. Our findings reveal that the prevalence rate of the virus, as determined by PCR detection, is 30.8% across all sampling sites, a result similar to the occurrence in the coastal Pacific herring obtained by a PCR-based approach^18^. The RNA-seq analysis provided strong evidence for the infection status of the positive subset: ENV-positive sampled fish showed high transcriptional activity of the virus, indicating active replication. This activity was demonstrated by thousands of reads mapping to 73 viral sequences, providing conclusive evidence of an active replication cycle and high biosynthetic activity within the host cell. In contrast, the host transcriptome profile notably showed no differential gene expression (DGE) between the ENV-positive and ENV-negative groups (FDR > 0.05), suggesting that the host immune system is not mounting a measurable, coordinated anti-viral response. Yet, this hypothesis needs to be interpreted cautiously, given the limitations of selecting samples based on PCR results. A qPCR might have been more sensitive and qualitative in distinguishing positive vs. negative samples. Given the absence of overt disease symptoms, future research should explore whether the detected viral replication reflects the presence of asymptomatic carriers or individuals predisposed to persistent infection. Further studies will also be needed to better understand the impact of ENV prevalence on herring populations and to elucidate how the virus progresses within its ecological niche. These studies will be essential for developing effective management strategies and for assessing the broader ecological implications of ENV in herring stocks.

### The case of *Ichthyophonus*

*Ichthyophonus hoferi* is recognized as a significant pathogen of teleost fish, known globally for causing widespread epizootics and resulting in substantial ecological losses in numerous marine and freshwater populations^5^. Among the most susceptible hosts is Atlantic herring *(Clupea harengus*), with this pathogen responsible for mass mortality due to the disease^5,21,22^. The presence of several reservoir hosts, including halibut *(Hippoglossus hippoglossus)*, flounder *(Pseudopleuronectes americanus)*, plaice *(Pleuronectes platessa)*, and rockfish *(Sebastes spp.)*^23^, facilitates the dissemination of this pathogen. However, the exact transmission mode remains hypothetical for *Clupea spp*^24^. Additionally, there is speculation about a calanoid copepod serving as an intermediate host^20^, but Kocan^24^ found no evidence to support this hypothesis. A study conducted in the spring of 2011 on Pacific herring reported a higher prevalence of infection associated with host size and age, along with observed seasonal and annual variations^25^. However, we did not observe any statistically significant difference in length, sex, or spawning period. This lack of observed correlation with abiotic or host factors may be attributed to the limitations of our field sampling model, specifically the small sample size of positive individuals (*n* = 21) and the low sensitivity of endpoint PCR to detect subtle variations in infection intensity.

Geographically, our results showed no significant correlation with sampling stations, even though the herrings likely schooled together before the fall spawning season. An important consideration is that Atlantic herring in the Gulf of St. Lawrence comprises both spring and fall spawning stocks that may differ genetically and ecologically. Although our results did not identify significant differences in pathogen prevalence between these groups, future work should treat stock identity explicitly when assessing disease dynamics and potential inter-stock transmission, as these components experience partly distinct life histories and habitat use.

The presence of parasitic DNA in the blood is likely derived from parasites that disseminate through the bloodstream and lymphatic system or their spores. It is important to remember, however, that the presence of *Ichthyophonus* DNA does not necessarily reflect active replication of the parasite. In contrast to ENV, which typically replicates directly in the blood cells (erythrocytes) of infected fish, *Ichthyophonus* replication is typically visceral, occurring primarily in various internal organs and tissues (e.g., liver, kidney, spleen, and muscles)^26^. Therefore, while parasite DNA is circulating, the RNA fragments derived from active replication are localized to these internal tissues and are consequently less likely to be detected in the blood sample. Future work will need to address whether *Ichthyophonus* DNA in the blood indicates the onset of a full-blown disease or a persistent infection.

### The impact on the circulating microbiome

Although the 16 S rRNA analyses revealed diverse bacterial communities in herring blood, we did not observe significant differences in alpha or beta diversity between ENV- or *Ichthyophonus*-positive and negative individuals. This finding suggests that these infections do not produce major dysbiosis in the circulating microbiome, at least at the subclinical stage detected in this study. Consequently, systemic viral or parasitic infections may have limited detectable effects on circulating microbial composition. Future longitudinal studies, ideally targeting both blood and tissue compartments, will be needed to determine whether more advanced or symptomatic infections exert a stronger influence on the host microbiome.

### The sampling and analysis platform

Our work showed that PCR and RNA-Seq methodologies using a single drop of blood preserved on FTA cards offer several significant advantages for managing sampling of wild fish populations. Methods for detecting pathogens have evolved considerably over the past few decades, from electron microscopic observations of the ENV virus^27^ to PCR-based methods^28^, to quantitative real-time PCR^29^ to an enriched method using Fluidigm BioMark Platform for High-Throughput Microbe Monitoring, which can detect as low as 40 copies per fluidic column^30^. Although FTA card-like media have been primarily used for RNA storage, particularly for body fluids, our results support previous studies showing that this matrix can also be used for high-throughput sequencing analyses of transcriptomes in blood cells and the detection of viral gene expression^31^. RNA-Seq approaches are increasingly used to determine the etiologic agents of certain diseases, or sometimes inadvertently, when studying the transcriptome^32,33^.

### Applications to the management of Atlantic herring

Our findings provide several direct applications for the management and conservation of Atlantic herring populations in the Gulf of St. Lawrence. Firstly, our study provides the first comprehensive diagnostic and ecological baseline for the presence of ENV and *Ichthyophonus hoferi* in this region, offering quantitative data that were previously unavailable to managers and researchers. Such information is essential for effective fishery management, as infectious diseases can strongly influence stock dynamics. It also provides a foundation for future temporal monitoring and for detecting shifts in infection rates that may signal ecological change. Secondly, the validated PCR assays and detection workflow, developed here and taking advantage of simplified logistics using FTA cards, could be integrated into existing surveillance programs and substantially reduce the costs and technical barriers associated with marine disease diagnostics. In addition to enabling simultaneous DNA, RNA, and pathogen analyses, FTA cards support the creation of long-term biobanks of field samples, facilitating retrospective studies of disease dynamics, population structure, and environmental change over time. Finally, by revealing the presence of persistent, non-pathogenic ENV carriers and quantifying *Ichthyophonus* prevalence, our results provide essential biological context for future stock assessments aimed at disentangling the relative contributions of disease, environmental variation, and demographic processes to herring population trends in the Gulf of St. Lawrence.

### Study ldimitations

A critical assessment of our findings requires acknowledging the limitations inherent to our experimental model and technical choices. Our primary constraint stems from the need for large-scale field sampling, which limits environmental control and experimental design. To maximize sample throughput (*n* = 136), we opted for traditional endpoint PCR on FTA cards, a decision prioritizing high-throughput, low-cost screening over quantitative resolution. This reliance on a binary presence/absence result limited our ability to detect subtle epidemiological correlations with host or abiotic factors, particularly given the small sample size used for the high-resolution RNA-seq and microbiome subsets. A qPCR might have been more sensitive and qualitative in distinguishing positive vs. negative samples. Consequently, the observed lack of statistical association should be interpreted with caution. Furthermore, interpreting low-level pathogen detection in RNA-seq data is complicated by the need to distinguish a genuine biological signal from methodological artifacts. While this observation may suggest that ENV prevalence is broader than estimated by standard PCR methods, the low read counts in PCR-negative samples could also reflect sequencing artifacts, such as index-hopping bias^34,35^. Additional studies employing complementary approaches will be valuable for clarifying the true extent of low-level viral replication in wild herring populations.

## Conclusion

In summary, our findings underscore the importance of comprehensive monitoring and research on both ENV and *Ichthyophonus* infections in Atlantic herring and other species. Together, our results reveal a widespread presence of ENV in Atlantic herring, while *Ichthyophonus* appears to have a more limited and sporadic presence. Continued investigation into these pathogens will be crucial for assessing their impacts on fish populations and the broader marine environment.

## Materials and methods

### Sampling

**Fig. 1:**
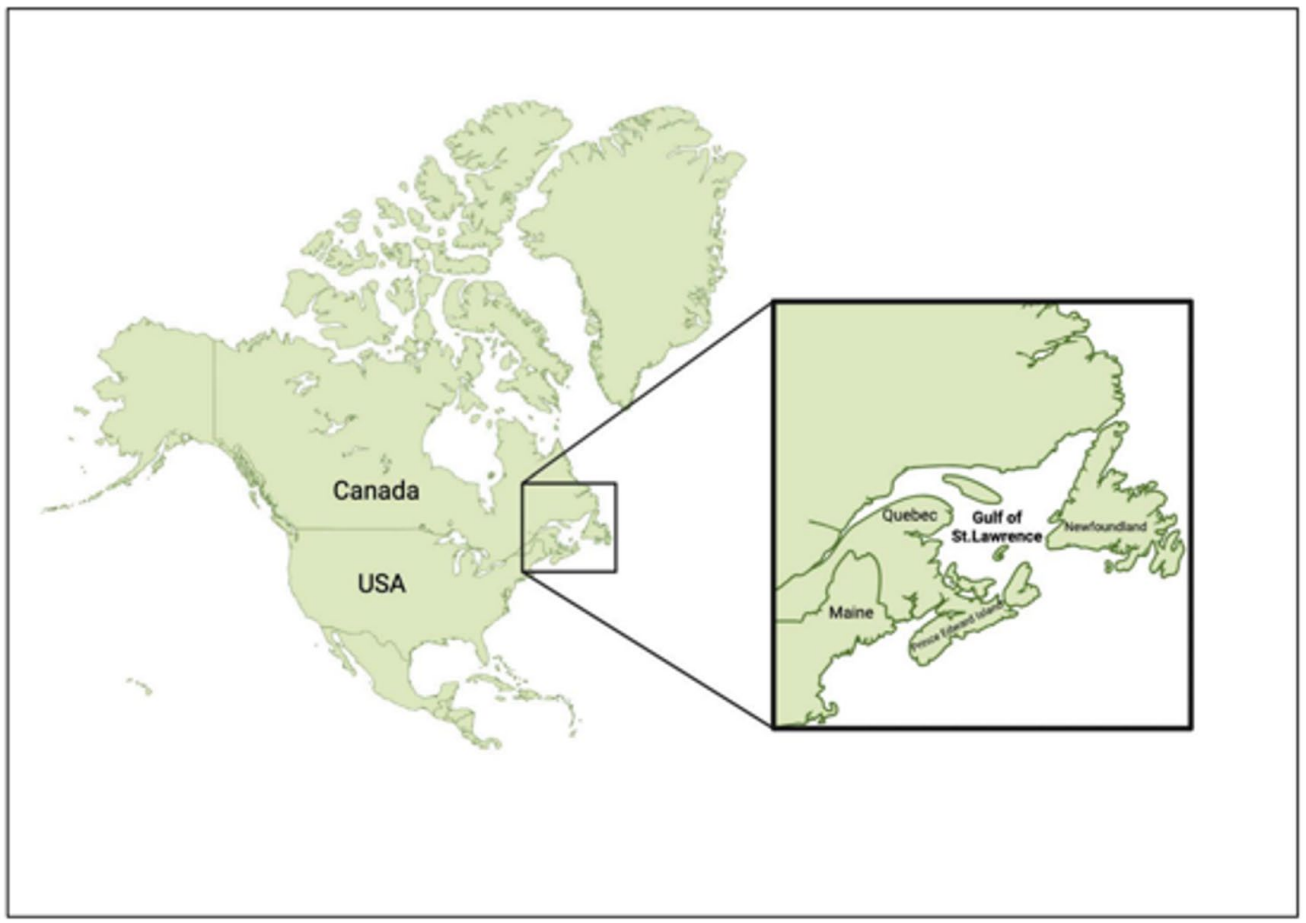
Sampling location in the Gulf of St. Lawrence. This map shows the geographical focus of our study. The Gulf of St. Lawrence is bordered by the provinces of Quebec and Newfoundland in Canada, as well as the northeastern United States.

Blood samples were taken from 38 Atlantic herring from the southern region (mean size: 274.9 ± 4.3 mm) and 144 individuals from the northern region (mean size: 284.3 ± 2.8 mm) of the Gulf of St. Lawrence. Located in Eastern Canada, the Gulf of St. Lawrence is one of the largest estuarine systems in the world that connects the St. Lawrence River to the Atlantic Ocean (Fig. 1). The sampling was conducted by Fisheries and Oceans Canada (DFO) from August 7 to September 30, 2023. Blood samples were collected immediately upon retrieval using heparin-coated 3-mL sterile syringes and 22-G needles. Drops of blood were immediately stored on Flinders Technology Associates (FTA™) cards (Sigma-Aldrich, Oakville, ON, Canada), allowed to air dry, and then stored in a plastic bag with a desiccant following the methodology of Fronton et al.^17^. Additionally, 140 fish from the northern group were dissected for detailed recording of sex, weight, maturity stage, gonad weight, and spawning group (Spring/Fall) (Supplementary Table 1). An additional 46 samples were collected from the southern Gulf of St. Lawrence between September 24 and October 4, 2024. All procedures adhered to the animal welfare laws, guidelines, and policies established by the Government of Canada, as approved by DFO, in accordance with ARRIVE guidelines.

### DNA extraction and screening for ENV and *Ichthyophonus*

All DNA procedures were conducted in a clean room with controlled pressure, temperature, and humidity, as outlined by Fronton et al.^17^. Individual 5.0 mm discs were punched from FTA cards, and total DNA was extracted using the QIAamp DNA Investigator Kit (Qiagen) according to the manufacturer’s instructions. DNA concentration was quantified using the Qubit dsDNA High Sensitivity (HS) assay (Invitrogen, CA, USA). PCR screening was performed for both pathogens with primers listed in Table 1. For ENV, the following amplification conditions were used: 35 cycles of denaturation at 95 °C for 30 s, annealing at 66 °C for 45 s, and extension at 72 °C for 45 s. Identical PCR conditions were applied for detecting *Ichthyophonus*, with the annealing temperature adjusted to 64 °C.

**Table 1:** Primers for molecular detection by PCR of ENV and *Ichthyophonus *sp. in herring.

Primers	NCBI Ref.	Gene	Sequences
Erythrocyte necrosis virus (ENV)			
VNE1F	KT211480.1	*Viral capsid*	5’-TACAAACCCTCATGGAGCCGACG − 3’
VNE1R	KT211480.1	*Viral capsid*	5’-ACACCCGTAAGCATAGGTGCGCG − 3’
VNE1aF	KT211480.1	*Viral capsid*	5’-CCTCACGGTCCTAATGAGCC-3’
VNE1aR	KT211480.1	*Viral capsid*	5’-CGCGTTCGATAGAAGGTCCA-3’
VNE1bF	KT211480.1	*Viral capsid*	5’-CCTCACGGTCCTAATGAGCCG-3’
VNE1bR	KT211480.1	*Viral capsid*	5’-CCGTAAGCATAGGTGCGCGTTC-3’
VNE1cF	KT211480.1	*Viral capsid*	5’-GGGCTGCATTTACAACGAGCGCA-3’
VNE1cR	KT211480.1	*Viral capsid*	5’-CGCGTTCGATAGAAGGTCCA-3’
*Ichthyophonus* sp.			
IchF1	KF987804	*5.8 S rRNA*	5’-ACCAGACACGCACTAACACG-3’ (a)
IchR1	KF987804	*5.8 S rRNA*	5’-ACTGGGCAATGGGTATCGTG-3’
Housekeeping gene			
EF1aSaltru164F	XM_029735647.1	*EF1a*	5’-ATTGCCACACTGCTCACATC-3’
EF1aSaltru164R	XM_029735647.1	*EF1a*	5’-CTGGAAGCTCTCCACACACA-3’

(a) From Hershberger et al.^36^.

### Blood microbiome analysis

Amplicon sequencing of the V3–V4 region of the 16S rRNA gene was conducted at the Centre d’Expertise et de Services Génome Québec (Montreal, QC, Canada) following a semi-nested PCR protocol. The first amplification utilized primers 27 F (5’-AGAGTTTGATCMTGGCTCAG-3’) and 805R (5′-GACTACHVGGGTATCTAATCC-3’), while the second amplification employed primers 341 F (5′-CCTACGGGNGGCWGCAG-3′) and 805R (5′-GACTACHVGGGTATCTAATCC-3’)^37^. Libraries were prepared using the TruSeq^®^ DNA Library Prep Kit (Illumina) and quantified with the KAPA Library Quantification Kit (Kapa Biosystems) before sequencing on the MiSeq platform (PE300) (MiSeq Reagent Kit v3, 600 cycles). FASTQ files were trimmed using Cutadapt (v2.8). Amplicon sequence variants (ASVs) were generated utilizing the DADA2 pipeline (v1.16.0)^38^ in R (v4.2.3; R Core Team, 2023). Forward and reverse reads were quality- filtered (maxEE), merged, and chimeras were removed, retaining sequences with an average quality score of ≥ 30. Taxonomic assignment was performed using the RDP Classifier (trainsetNo19)^39^. Microbial community analyses were conducted using phyloseq (v1.40.0)^40^ and DESeq2 (v1.36.0)^41^. Raw reads were deposited in the NCBI Sequence Read Archive (SRA) under accession number PRJNA1258723.

### Transcriptomics

Total RNA was extracted from blood spots on FTA cards using TRIzol™ Reagent (Invitrogen, CA, USA), followed by purification with the RNeasy Plus Mini Kit (Qiagen). RNA concentration was measured using the Qubit™ RNA High Sensitivity (HS) assay (Life Technologies), and RNA quality was assessed with a Nanodrop spectrophotometer. Depleted rRNA libraries were prepared at Genome Quebec (Montreal, QC, Canada) using the NEB Stranded Library Prep Kit for Illumina. Sequencing was performed on a NovaSeq 6000 platform (Illumina) with 100 bp paired-end reads, yielding between 33,670,009 and 86,489,303 reads per sample with sequence lengths ranging from 49 to 101 base pairs and GC contents between 43% and 45%. The raw reads underwent quality filtering and adapter trimming using Trimmomatic 0.39^42^. Raw reads were evaluated using FastQC, and quality metrics indicated high-quality sequencing outputs across all samples, with no sequences flagged as poor quality (Supplementary Table **S1**). The processed reads were subsequently mapped to the Atlantic herring genome using STAR 2.7.11a^43^. Mapping statistics, including the proportion of uniquely mapped reads and the percentage of reads assigned to genes. Transcript decontamination and normalization were conducted with DADA2 (v1.30.0; Callahan et al., 2016). Differential expression analysis was performed using DESeq2 (v1.36.0)^41^ within the R environment (v4.2.3), with genes considered significantly differentially expressed if they had a p-value ≤ 0.01 and a log2 fold change between − 1.57 and 1.57. For the transcriptome analysis of the positive and negative ENV groups, differential expression was similarly analyzed using DESeq2, with the same criteria for significance applied. Raw reads were deposited in the NCBI Sequence Read Archive (SRA) under accession number PRJNA1258236.

### Statistical analyses

Statistical analyses were performed to assess the differential microbiome associated with ENV and *Ichthyophonus*, and to explore correlations with biological factors, including length, sex, and age. The relationships between categorical variables were evaluated using Pearson’s Chi-squared test, while logistic regression was employed to determine the influence of biological factors on microbial diversity and infection status. Proportions tests were conducted to compare group proportions across different categories. Additionally, because temperature, oxygen, depth and salinity values did not follow a normal distribution with respect to ENV and *Ichthyophonus* status, comparisons were performed using the Wilcoxon rank-sum test. The spatial relationships among samples were further analyzed using the spdep package (v1.2–7)^44^, with the neighborhood structure defined by k = 5 nearest neighbors. A measure of the overall clustering of spatial data was carried out using Moran’s *I*method^45,36^.

## Supplementary Information

Below is the link to the electronic supplementary material.

Supplementary Material 1

Supplementary Material 2

## Data Availability

The raw sequencing data for the transcriptomic analyses generated in this study have been deposited in the NCBI Sequence Read Archive (SRA) under accession number PRJNA1258723. All other relevant data supporting the findings of this study are available within the article and its Supplementary Information files.
