# Supplementary material for: Comprehensive pathogen diagnostics in wild fish populations using blood-based molecular strategies: an Atlantic herring case study
Source: Sci Rep. 2025 Dec 31;16:34. doi: 10.1038/s41598-025-28653-8 (PMC12764890; doi:10.1038/s41598-025-28653-8)
Supplement: Supplementary file 1 — Supplementary Material 1 [file 41598_2025_28653_MOESM1_ESM.pdf]

Supplementary Information

Detection of Viral and Parasitic Pathogens in Atlantic Herring *(Clupea harengus)*

France Caza[1], Fanny Fronton[1], Lina Ennia[1],

Dominique Robert[2], Yves St-Pierre[1]

1) INRS-Center Armand-Frappier Santé Technologie,

531 Boul. des Prairies, Laval, QC, Canada, H7V 1B7

2) Institut des Sciences de la Mer, Université du Québec à Rimouski,

310, allée des Ursulines, C.P. 3300 Rimouski, QC, Canada, G5L 3A1

Corresponding Author

Yves St-Pierre, Ph.D.
Professor
Institut National de la Recherche Scientifique (INRS)
INRS-Centre Armand-Frappier Santé Technologie.
Pasteur Institute International Network
531 Boul. Des Prairies,
Laval, Québec, Canada, H7V 1B7.
Phone : 450-686-5354
E-mail: yves.st-pierre@inrs.ca

Figure S1

```
>Erythrocytic necrosis virus isolate SEQ_86 major capsid protein mRNA,
partial cds
Sequence ID: MK638677.1 Length: 1326
Range 1: 420 to 730
Score:564 bits(305), Expect:3e-156,

Identities:309/311(99%),  Gaps:0/311(0%), Strand: Plus/Plus

Query  26   CTGGGCTGCATTTACAACGAGCGCAAGTAAAAAGAGCGGTTACGATACAATGATTGGAAA  85
            ||||||||||||||||||||||||||||||||||||||||||||||||||||||||||||
Sbjct  420  CTGGGCTGCATTTACAACGAGCGCAAGTAAAAAGAGCGGTTACGATACAATGATTGGAAA  479

Query  86   TGTAGACGACCTCATTTCTCCTCACGGTCCTAATGAGCCGTTGAAATCCAAGATTCTCAA  145
            ||||||||||||||||||||||||||||||||||||||||||||||||||||||||||||
Sbjct  480  TGTAGACGACCTCATTTCTCCTCACGGTCCTAATGAGCCGTTGAAATCCAAGATTCTCAA  539

Query  146  ACTTCCTATTCCTTTCTTTTTCTCCCGTGACTCTGGAATAGCTCTTCCCACTGGTGCTCT  205
             |||||||||||||||||||||||||||||||||||||||||||||||||||||||||||
Sbjct  540  TCTTCCTATTCCTTTCTTTTTCTCCCGTGACTCTGGAATAGCTCTTCCCACTGGTGCTCT  599

Query  206  CTTGTATACTGAGACCAGAATTAGTTTTAAGCTCAGGAATTGGAATCAGCTCTTGATTCT  265
            | ||||||||||||||||||||||||||||||||||||||||||||||||||||||||||
Sbjct  600  CCTGTATACTGAGACCAGAATTAGTTTTAAGCTCAGGAATTGGAATCAGCTCTTGATTCT  659

Query  266  TGAGAATGCCAATCCACTTCCCAATACTCCCAATGGTGGAGTTCCTATGGTTGGACCTTC  325
            ||||||||||||||||||||||||||||||||||||||||||||||||||||||||||||
Sbjct  660  TGAGAATGCCAATCCACTTCCCAATACTCCCAATGGTGGAGTTCCTATGGTTGGACCTTC  719

Query  326  TATCGAACGCG  336
            |||||||||||
Sbjct  720  TATCGAACGCG  730
```

Figure S1: DNA Sequence Alignment of Erythrocytic Necrosis Virus Isolate Capsid Protein
Results of DNA sequencing for the major capsid protein mRNA of ENV. The sequence alignment is shown between the query PCR-generated amplicons and the subject sequence from the database with Sequence ID MK638677.

Figure S2

```
Sequence ID: GQ402856.1 Length: 734
Range 1: 60 to 680
Score:933 bits(505), Expect:0.0,
Identities:585/623(94%),  Gaps:8/623(1%), Strand: Plus/Plus

Query  12   AAA-CC-ACCCCGGCCTGATGGGAAACCAACAGTGCCATTATTAACAACAACCGTTATGT  69
            ||| || |||||||||||||||||||||||||||||||||||||||||||||||||||||
Sbjct  60   AAACCCAACCCCGGCCTGATGGGAAACCAACAGTGCCATTATTAACAACAACCGTTATGT  119

Query  70   CTTGTTTACTAGTAGGTAATTGAGCTAATTTTTGGCACTTTTACCTATTGGTTTTAAGAC  129
            | ||||||||||||||||||||||||||||||||||||||||||||||||||||||||||
Sbjct  120  CGTGTTTACTAGTAGGTAATTGAGCTAATTTTTGGCACTTTTACCTATTGGTTTTAAGAC  179

Query  130  ACGACAGTTTTATTATAAAATATATACCCCATTAAAAACAATCTGGCGGCTTGATGCTaa  189
            |||||||||||||||||||||||||||||||||||||||||||||| |||||||||||||
Sbjct  180  ACGACAGTTTTATTATAAAATATATACCCCATTAAAAACAATCTGGTGGCTTGATGCTAA  239

Query  190  aaaacaaaaaaaTTTAAACAACTTTTAACGGTGGATCTCTAGGCTCGTGCATCGATGAAA  249
            |||| ||||||||||| |||||||||||||||||||||||||||||||||||||||||| 
Sbjct  240  AAAA-AAAAAAATTTAGACAACTTTTAACGGTGGATCTCTAGGCTCGTGCATCGATGAAG  298

Query  250  AACGCAGCGAAATGCGATACGTAGTGTGAATTGCAAATCTTCGTGAATCATCGAATCTTT  309
            |||||||||||||||||||||||||||||||||| |||||||||||||||||||||||||
Sbjct  299  AACGCAGCGAAATGCGATACGTAGTGTGAATTGCAGATCTTCGTGAATCATCGAATCTTT  358

Query  310  GAACGCAAATTGCGGTTCCAGGTAACTCCTGGGACCATGCCTGGTTGAGTGTCATCAAAA  369
            ||||||||||||||||||||||||||||||||||||||||||||||||||||||||||||
Sbjct  359  GAACGCAAATTGCGGTTCCAGGTAACTCCTGGGACCATGCCTGGTTGAGTGTCATCAAAA  418

Query  370  CCCAACACTATATGTGGAATAAATGAGGTTCACGGTGaaaaaaaa--aCTTGTATTTTTG  427
            |||||||||||||||||||||||||| ||||||||||||||||||  |||||||||||| 
Sbjct  419  CCCAACACTATATGTGGAATAAATGAAGTTCACGGTGAAAAAAAAATACTTGTATTTTTT  478

Query  428  T--AACGGGGTGCCTTTGATTATAAGTCAAAGAAATTGCCTTTTCTTGGCTTGACTCACC  485
            |  ||| |||||| ||||| |||| ||||||| |||||||||||| |||||| ||||| |
Sbjct  479  TGGAACTGGGTGCTTTTGAATATAGGTCAAAGTAATTGCCTTTTCATGGCTTTACTCATC  538

Query  486  ATACAATGTTTTTACTGGCAAGTAATTGTTTTGTAAATAAAATCTGTGAGGAAAAAATGC  545
            |||| |||||||||||||||| | |||||||||||||| | |||||||| || |||||||
Sbjct  539  ATACGATGTTTTTACTGGCAATTTATTGTTTTGTAAATTATATCTGTGAAGAGAAAATGC  598

Query  546  AATGATTaaaaaaaaTAATAATACTACACATTAAACAAAGTAATGTTTTCTTTGATACCA  605
            |||||  ||||  ||||||||||||| |||||||||||||||||||||||||||| ||||
Sbjct  599  AATGA-AAAAAGTAATAATAATACTAGACATTAAACAAAGTAATGTTTTCTTTGAAACCA  657

Query  606  ACCACATTATTATAAAAAATTTG  628
            |  |||||||||||| |||||||
Sbjct  658  AAGACATTATTATAAGAAATTTG  680
```

Figure S2: DNA Sequence Alignment of Ichthyophonus-specific PCR Amplicons. DNA sequence alignment results for PCR amplicons generated using specific primers targeting Ichthyophonus. The alignment compares the query sequences (PCR amplicons) to a reference sequence from the database (Sequence ID: GQ402856.1).

Figure S3

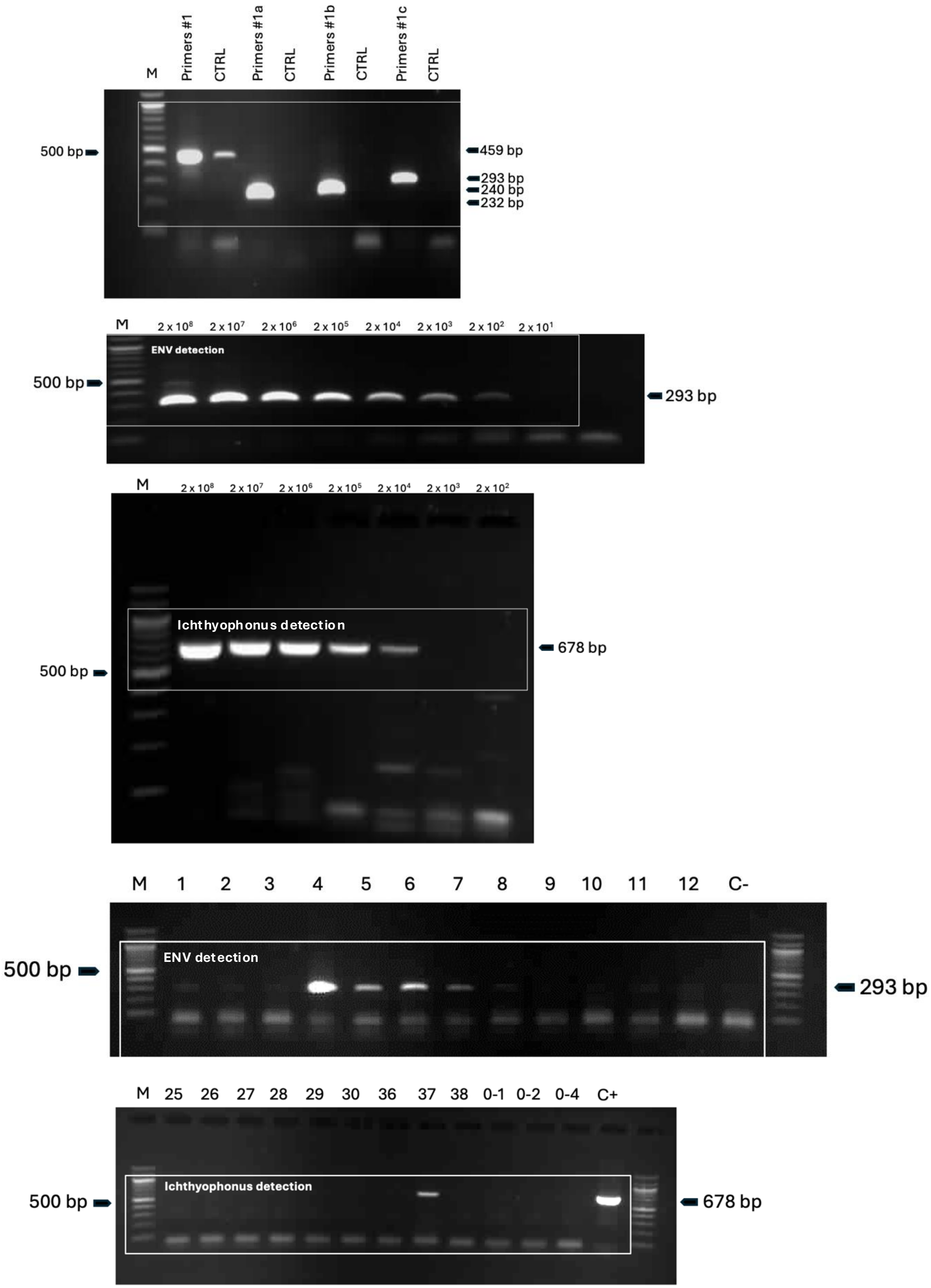

Figure S3: Original uncropped agarose gels for Extended Data Fig. 2a–c. Boxes indicate the cropped regions.

Supplementary Table S1

Table S1: Overview of RNA-Seq Read Statistics for Negative (Neg) and Positive (Pos) Samples.

Sample	Total Reads	Unique Reads	Unique Read (%)	Total Reads assigned	% of Total Reads Assigned
Neg15_5_PE	1,146,770	944,843	82.39	183,023	16.0
Neg20_PE	65,144,026	53,053,375	81.44	9,169,668	14.1
Neg21_PE	51,265,679	38,163,315	74.44	5,863,703	11.4
Neg29_PE	33,490,139	27,179,253	81.16	4,982,519	14.9
Neg57_1_PE	72,644,519	58,928,059	81.12	11,564,870	15.9
Pos32_PE	66,543,858	53,142,089	79.86	9,783,347	14.7
Pos4_PE	42,469,890	34,771,806	81.87	6,233,178	14.7
Pos53_2_PE	63,631,886	51,068,283	80.26	9,008,908	14.2
Pos6_PE	86,269,479	69,284,423	80.31	15,209,838	17.6
Pos33_PE	57,705,655	48,182,000	83.5	8,932,887	15.5
