# Supplementary material for: Comprehensive pathogen diagnostics in wild fish populations using blood-based molecular strategies: an Atlantic herring case study
Source: Sci Rep. 2025 Dec 31;16:34. doi: 10.1038/s41598-025-28653-8 (PMC12764890; doi:10.1038/s41598-025-28653-8)
Supplement: Supplementary file 2 — Supplementary Material 2 [file 41598_2025_28653_MOESM2_ESM.pdf]

Supplementary Table 1: Environmental and Biological Data of Herring Samples Collected Across Various Stations.

Sample	Date	ENV	Ichty	Sex	Length (mm)	Weight (g)	Age	Gon.weight (g)	Maturity	Spawn Group	Station	Latitude	Longitude	Temperature (C°)	Salinity	Oxygene	Depth (m)
1	2023-09-25	Neg	Neg	NA	245	NA	NA	NA	NA	NA	WM6O	48.0101	-64.81855	13.12	27.6	5.25	22
2	2023-09-25	Pos	Neg	NA	295	NA	NA	NA	NA	NA	WM6O	48.0101	-64.81855	13.12	27.6	5.25	22
3	2023-09-25	Neg	Neg	NA	250	NA	NA	NA	NA	NA	WM6O	48.0101	-64.81855	13.12	27.6	5.25	22
4	2023-09-25	Pos	Neg	NA	245	NA	NA	NA	NA	NA	WM6O	48.0101	-64.81855	13.12	27.6	5.25	22
5	2023-09-25	Pos	Neg	NA	240	NA	NA	NA	NA	NA	WM6O	48.0101	-64.81855	13.12	27.6	5.25	22
6	2023-09-25	Pos	Neg	NA	260	NA	NA	NA	NA	NA	WM6O	48.0101	-64.81855	13.12	27.6	5.25	22
7	2023-09-25	Pos	Neg	NA	250	NA	NA	NA	NA	NA	WM6O	48.0101	-64.81855	13.12	27.6	5.25	22
8	2023-09-25	Pos	Pos	NA	245	NA	NA	NA	NA	NA	WM6O	48.0101	-64.81855	13.12	27.6	5.25	22
9	2023-09-26	Neg	Neg	NA	285	NA	NA	NA	NA	NA	WM6O	48.0101	-64.81855	13.12	27.6	5.25	22
11	2023-09-26	Pos	Pos	NA	280	NA	NA	NA	NA	NA	M7I	47.83563	-65.07716	11.52	25.78	4.85	28
13	2023-09-26	Neg	Neg	NA	310	NA	NA	NA	NA	NA	M7I	47.83563	-65.07716	11.52	25.78	4.85	28
14	2023-09-26	Neg	Neg	NA	295	NA	NA	NA	NA	NA	M7I	47.83563	-65.07716	11.52	25.78	4.85	28
15	2023-09-26	Neg	Neg	NA	265	NA	NA	NA	NA	NA	M7I	47.83563	-65.07716	11.52	25.78	4.85	28
16	2023-09-26	Neg	Neg	NA	305	NA	NA	NA	NA	NA	M7I	47.83563	-65.07716	11.52	25.78	4.85	28
17	2023-09-26	Neg	Neg	NA	310	NA	NA	NA	NA	NA	M7I	47.83563	-65.07716	11.52	25.78	4.85	28
18	2023-09-26	Neg	Pos	NA	275	NA	NA	NA	NA	NA	N14I	47.77786	-65.29126	7.99	27.76	4.82	32
19	2023-09-26	Neg	Neg	NA	240	NA	NA	NA	NA	NA	N14I	47.77786	-65.29126	7.99	27.76	4.82	32
20	2023-09-26	Neg	Neg	NA	240	NA	NA	NA	NA	NA	N14I	47.77786	-65.29126	7.99	27.76	4.82	32
21	2023-09-26	Neg	Neg	NA	240	NA	NA	NA	NA	NA	N14I	47.77786	-65.29126	7.99	27.76	4.82	32
22	2023-09-27	Neg	Neg	NA	245	NA	NA	NA	NA	NA	SE6I	47.85783	-64.25892	4.91	30.9	6.31	46
23	2023-09-27	Pos	Neg	NA	250	NA	NA	NA	NA	NA	SE6I	47.85783	-64.25892	4.91	30.9	6.31	46
24	2023-09-27	Neg	Pos	NA	245	NA	NA	NA	NA	NA	SE6I	47.85783	-64.25892	4.91	30.9	6.31	46
25	2023-09-27	Neg	Neg	NA	260	NA	NA	NA	NA	NA	SE6I	47.85783	-64.25892	4.91	30.9	6.31	46
26	2023-09-29	Pos	Neg	NA	245	NA	NA	NA	NA	NA	WM10I	47.97689	-64.60594	9.13	28.83	5.3	33
27	2023-09-29	Pos	Neg	NA	260	NA	NA	NA	NA	NA	WM10I	47.97689	-64.60594	9.13	28.83	5.3	33
28	2023-09-29	Neg	Neg	NA	310	NA	NA	NA	NA	NA	WM10I	47.97689	-64.60594	9.13	28.83	5.3	33
29	2023-09-30	Neg	Neg	NA	300	NA	NA	NA	NA	NA	NW3I	48.08094	-64.10161	7.92	29.94	5.84	31
30	2023-09-30	Neg	Neg	NA	295	NA	NA	NA	NA	NA	NW3I	48.08094	-64.10161	7.92	29.94	5.84	31
32	2023-09-30	Pos	Neg	NA	290	NA	NA	NA	NA	NA	NW3I	48.08094	-64.10161	7.92	29.94	5.84	31
33	2023-09-30	Pos	Neg	NA	310	NA	NA	NA	NA	NA	NW3I	48.08094	-64.10161	7.92	29.94	5.84	31
35	2023-09-30	Pos	Pos	NA	305	NA	NA	NA	NA	NA	NW3I	48.08094	-64.10161	7.92	29.94	5.84	31
36	2023-09-30	Pos	Neg	NA	295	NA	NA	NA	NA	NA	NW3I	48.08094	-64.10161	7.92	29.94	5.84	31
37	2023-09-30	Neg	Pos	NA	285	NA	NA	NA	NA	NA	NW3I	48.08094	-64.10161	7.92	29.94	5.84	31
38	2023-09-30	Pos	Neg	NA	285	NA	NA	NA	NA	NA	NW3I	48.08094	-64.10161	7.92	29.94	5.84	31
10_1	2023-09-30	Neg	Neg	F	310	272	8	3.62	3	P	10	48.10766667	-59.53933333	6.21	34.25	159.73	202
10_2	2023-08-07	Neg	Neg	M	300	272	7	2.14	8	A	10	48.10766667	-59.53933333	6.21	34.25	159.73	202
10_3	2023-08-07	Pos	Neg	M	310	268	10	2.44	3	A	10	48.10766667	-59.53933333	6.21	34.25	159.73	202
10_4	2023-08-07	Pos	Neg	M	350	404	8	77.6	5	A	10	48.10766667	-59.53933333	6.21	34.25	159.73	202
10_5	2023-08-07	Pos	Neg	F	283	NA	6	2.6	8	A	10	48.10766667	-59.53933333	6.21	34.25	159.73	202
11_1	2023-08-07	Pos	Neg	NA	320	269.5	NA	NA	NA	NA	11	48.088	-59.41233333	3.41	33.21	237.28	145
11_2	2023-08-07	Neg	Neg	NA	310	295	NA	NA	NA	NA	11	48.088	-59.41233333	3.41	33.21	237.28	145
11_3	2023-08-07	Neg	Neg	NA	300	270.5	NA	NA	NA	NA	11	48.088	-59.41233333	3.41	33.21	237.28	145
11_4	2023-08-07	Neg	Neg	NA	277	202	NA	NA	NA	NA	11	48.088	-59.41233333	3.41	33.21	237.28	145
11_5	2023-08-07	Neg	Neg	NA	269	191	NA	NA	NA	NA	11	48.088	-59.41233333	3.41	33.21	237.28	145
13_1	2023-08-07	Pos	Neg	M	290	196	5	33.8	4	P	13	48.27283333	-59.4355	1.38	32.43	298.72	113
13_2	2023-08-07	Pos	Neg	F	292	180	4	2.26	8	P	13	48.27283333	-59.4355	1.38	32.43	298.72	113
13_3	2023-08-07	Pos	Neg	M	301	187	6	1	7	P	13	48.27283333	-59.4355	1.38	32.43	298.72	113
13_4	2023-08-07	Pos	Neg	M	295	188	6	1.38	8	P	13	48.27283333	-59.4355	1.38	32.43	298.72	113
13_5	2023-08-07	Neg	Neg	F	262	124	3	0.88	2	P	13	48.27283333	-59.4355	1.38	32.43	298.72	113
15_1	2023-08-07	Neg	Neg	M	293	172	6	0.92	7	P	15	48.33933333	-59.235	1.72	32.39	276.48	87
15_2	2023-08-07	Pos	Neg	M	300	187	5	0.86	8	P	15	48.33933333	-59.235	1.72	32.39	276.48	87
15_3	2023-08-07	Pos	Neg	F	291	175	6	1.46	8	A	15	48.33933333	-59.235	1.72	32.39	276.48	87
15_4	2023-08-07	Neg	Neg	M	280	144	6	0.68	7	A	15	48.33933333	-59.235	1.72	32.39	276.48	87
15_5	2023-08-07	Neg	Pos	F	276	162	6	1.18	7	A	15	48.33933333	-59.235	1.72	32.39	276.48	87
16_1	2023-08-08	Neg	Neg	NA	291	172	NA	NA	NA	NA	16	48.429	-59.1425	1.5	32.17	292.03	75
16_2	2023-08-08	Neg	Neg	NA	281	147	NA	NA	NA	NA	16	48.429	-59.1425	1.5	32.17	292.03	75
16_3	2023-08-08	Pos	Neg	NA	293	178	NA	NA	NA	NA	16	48.429	-59.1425	1.5	32.17	292.03	75
16_4	2023-08-08	Neg	Pos	NA	275	152.5	NA	NA	NA	NA	16	48.429	-59.1425	1.5	32.17	292.03	75
16_5	2023-08-08	Pos	Neg	NA	285	138	NA	NA	NA	NA	16	48.429	-59.1425	1.5	32.17	292.03	75
21_1	2023-08-17	Neg	Neg	F	300	162.5	6	7.14	7	P	21	49.13233333	-59.52983333	6.99	34.67	94.06	242
21_2	2023-08-17	Neg	Neg	F	281	151	6	1.22	8	A	21	49.13233333	-59.52983333	6.99	34.67	94.06	242
21_3	2023-08-17	Neg	Neg	M	289	164	6	29.86	4	A	21	49.13233333	-59.52983333	6.99	34.67	94.06	242
21_4	2023-08-17	Pos	Neg	F	286	133.5	6	1.14	8	P	21	49.13233333	-59.52983333	6.99	34.67	94.06	242
21_5	2023-08-17	Neg	Neg	M	252	116	2	0.36	2	A	21	49.13233333	-59.52983333	6.99	34.67	94.06	242
53_1	2023-08-25	Neg	Neg	M	310	192	6	1.44	8	A	53	49.53383333	-59.812	6.98	34.65	82.34	245
53_2	2023-08-25	Pos	Pos	M	286	146	5	0.74	7	P	53	49.53383333	-59.812	6.98	34.65	82.34	245

53_3	2023-08-25	Pos	Neg	F	296	186	5	2.22	8	A	53	49.53383333	-59.812	6.98	34.65	82.34	245
53_4	2023-08-25	Pos	Neg	F	296	182	6	2.72	3	A	53	49.53383333	-59.812	6.98	34.65	82.34	245
53_5	2023-08-25	Pos	Neg	M	285	170	6	1.76	3	A	53	49.53383333	-59.812	6.98	34.65	82.34	245
54_1	2023-08-25	Neg	Neg	M	305	192	9	4.36	3	P	54	49.54783333	-59.2825	6.93	34.62	90.07	233
54_2	2023-08-25	Neg	Neg	M	303	160	6	1.18	7	A	54	49.54783333	-59.2825	6.93	34.62	90.07	233
54_5	2023-08-25	Neg	Pos	F	305	168	6	1.78	8	P	54	49.54783333	-59.2825	6.93	34.62	90.07	233
55_1	2023-08-25	Neg	Neg	F	254	120	3	4.76	3	A	55	49.5345	-58.92766667	5.99	34.15	122.87	173
55_2	2023-08-25	Pos	Neg	M	360	344	10	66.82	4	P	55	49.5345	-58.92766667	5.99	34.15	122.87	173
55_3	2023-08-25	Neg	Neg	M	285	142	6	1.12	7	A	55	49.5345	-58.92766667	5.99	34.15	122.87	173
55_4	2023-08-25	Neg	Neg	F	283	134	6	1.36	7	A	55	49.5345	-58.92766667	5.99	34.15	122.87	173
55_5	2023-08-25	Neg	Neg	F	365	366	10	46.44	4	A	55	49.5345	-58.92766667	5.99	34.15	122.87	173
56_1	2023-08-25	Neg	Neg	M	297	174	5	1.7	8	P	56	49.67533333	-58.6475	3.66	33.29	198.8	145
56_3	2023-08-25	Neg	Neg	F	296	182	6	2.6	8	A	56	49.67533333	-58.6475	3.66	33.29	198.8	145
56_4	2023-08-25	Neg	Pos	M	305	196	5	1.2	8	P	56	49.67533333	-58.6475	3.66	33.29	198.8	145
56_5	2023-08-25	Neg	Neg	F	299	172	6	1.62	8	P	56	49.67533333	-58.6475	3.66	33.29	198.8	145
57_1	2023-08-25	Neg	Neg	M	283	150	6	0.74	7	P	57	49.65666667	-58.37433333	1.18	32.45	279.58	86
57_2	2023-08-25	Neg	Neg	F	302	166	6	1.36	7	P	57	49.65666667	-58.37433333	1.18	32.45	279.58	86
57_3	2023-08-25	Pos	Neg	F	281	144	6	0.94	7	A	57	49.65666667	-58.37433333	1.18	32.45	279.58	86
57_4	2023-08-25	Pos	Neg	F	292	170	6	0.94	8	A	57	49.65666667	-58.37433333	1.18	32.45	279.58	86
61_1	2023-08-26	Pos	Neg	F	281	160	7	1.58	8	P	61	50.14466667	-57.88233333	5.92	34.13	114.36	174
61_2	2023-08-26	Pos	Neg	M	327	258	8	6.18	3	A	61	50.14466667	-57.88233333	5.92	34.13	114.36	174
61_3	2023-08-26	Neg	Neg	M	381	382	7	6.14	3	A	61	50.14466667	-57.88233333	5.92	34.13	114.36	174
61_4	2023-08-26	Neg	Neg	F	286	156	6	1.5	8	A	61	50.14466667	-57.88233333	5.92	34.13	114.36	174
61_5	2023-08-26	Neg	Neg	F	280	148	6	1.74	8	A	61	50.14466667	-57.88233333	5.92	34.13	114.36	174
62_1	2023-08-26	Neg	Neg	F	312	200	7	2.7	8	P	62	50.20316667	-57.97116667	6.95	34.6	69.56	226
62_2	2023-08-26	Neg	Neg	F	306	202	4	28.36	4	A	62	50.20316667	-57.97116667	6.95	34.6	69.56	226
62_3	2023-08-26	Neg	Neg	F	306	184	6	3.18	3	A	62	50.20316667	-57.97116667	6.95	34.6	69.56	226
62_4	2023-08-26	Pos	Neg	F	301	300	6	5.38	3	A	62	50.20316667	-57.97116667	6.95	34.6	69.56	226
62_5	2023-08-26	Pos	Neg	NA	369	334	8	0.16	NA	A	62	50.20316667	-57.97116667	6.95	34.6	69.56	226
63_1	2023-08-26	Neg	Neg	F	290	170	6	1.38	8	P	63	50.391	-57.73416667	6.85	34.55	65.57	230
63_2	2023-08-26	Neg	Pos	M	291	172	6	1.56	8	A	63	50.391	-57.73416667	6.85	34.55	65.57	230
63_4	2023-08-26	Neg	Neg	F	295	180	6	2.5	8	P	63	50.391	-57.73416667	6.85	34.55	65.57	230
63_5	2023-08-26	Neg	Neg	F	305	192	6	3.04	3	P	63	50.391	-57.73416667	6.85	34.55	65.57	230
64_2	2023-08-26	Neg	Pos	F	289	NA	6	1.6	8	P	64	50.57483333	-57.48333333	3.57	33.25	199.27	136
64_4	2023-08-26	Neg	Neg	F	247	NA	3	0.84	7	P	64	50.57483333	-57.48333333	3.57	33.25	199.27	136
64_5	2023-08-26	Neg	Neg	F	285	NA	6	1.62	7	P	64	50.57483333	-57.48333333	3.57	33.25	199.27	136
65_1	2023-08-26	Neg	Neg	M	248	92	3	0.12	2	A	65	50.63716667	-57.67016667	6.94	34.6	64.02	255
65_2	2023-08-26	Neg	Neg	M	290	170	6	1.38	3	P	65	50.63716667	-57.67016667	6.94	34.6	64.02	255
65_3	2023-08-26	Neg	Neg	F	348	284	6	28.8	4	A	65	50.63716667	-57.67016667	6.94	34.6	64.02	255
65_4	2023-08-26	Neg	Neg	F	310	196	5	2.02	8	P	65	50.63716667	-57.67016667	6.94	34.6	64.02	255
65_5	2023-08-26	Neg	Neg	M	307	186	4	25.44	4	A	65	50.63716667	-57.67016667	6.94	34.6	64.02	255
66_1	2023-08-26	Neg	Pos	F	279	142	5	0.88	8	P	66	50.73616667	-57.643	1.92	32.77	252.59	120
66_3	2023-08-26	Neg	Neg	M	283	156	6	0.84	8	P	66	50.73616667	-57.643	1.92	32.77	252.59	120
66_4	2023-08-26	Neg	Neg	M	245	90	2	0.06	2	A	66	50.73616667	-57.643	1.92	32.77	252.59	120
66_5	2023-08-26	Neg	Neg	F	221	74	3	0.44	2	P	66	50.73616667	-57.643	1.92	32.77	252.59	120
70_1	2023-08-27	Neg	Pos	M	296	172	5	1.32	8	P	70	50.56666667	-58.13333333	5.05	33.79	143.97	163
70_3	2023-08-27	Neg	Neg	F	296	160	6	0.38	7	P	70	50.56666667	-58.13333333	5.05	33.79	143.97	163
70_4	2023-08-27	Neg	Neg	F	296	156	5	0.84	7	A	70	50.56666667	-58.13333333	5.05	33.79	143.97	163
70_5	2023-08-27	Neg	Neg	F	337	252	6	5.02	3	P	70	50.56666667	-58.13333333	5.05	33.79	143.97	163
76_1	2023-08-28	Neg	Pos	F	331	252	6	2.24	8	P	76	49.8435	-59.0715	6.92	34.59	76.33	221
76_2	2023-08-28	Neg	Neg	F	331	224	6	4.28	3	A	76	49.8435	-59.0715	6.92	34.59	76.33	221
76_3	2023-08-28	Neg	Neg	M	300	216	6	41.38	4	A	76	49.8435	-59.0715	6.92	34.59	76.33	221
76_4	2023-08-28	Neg	Neg	M	286	146	6	0.8	8	P	76	49.8435	-59.0715	6.92	34.59	76.33	221
76_5	2023-08-28	Neg	Neg	M	298	180	6	1.34	8	P	76	49.8435	-59.0715	6.92	34.59	76.33	221
100_1	2023-08-31	Neg	Neg	M	306	204	5	26.9	4	A	100	49.9085	-64.7855	3.82	33.39	150.49	155
100_3	2023-08-31	Neg	Neg	F	268	164	8	3.18	8	A	100	49.9085	-64.7855	3.82	33.39	150.49	155
100_4	2023-08-31	Neg	Neg	F	262	130	2	8	3	A	100	49.9085	-64.7855	3.82	33.39	150.49	155
100_5	2023-08-31	Neg	Neg	M	267	154	7	8.56	3	A	100	49.9085	-64.7855	3.82	33.39	150.49	155
102_1	2023-08-31	Neg	Neg	M	251	110	3	5.48	3	A	102	50.10216667	-65.0115	3.73	33.16	135.58	143
102_3	2023-08-31	Neg	Neg	F	230	86	2	0.36	2	A	102	50.10216667	-65.0115	3.73	33.16	135.58	143
102_5	2023-08-31	Neg	Neg	F	178	42	2	0.08	1	A	102	50.10216667	-65.0115	3.73	33.16	135.58	143
111_1	2023-09-01	Neg	Neg	M	266	154	3	22.3	4	A	111	49.71866667	-66.99316667	4.79	33.83	105.45	171
111_4	2023-09-01	Neg	Neg	M	266	132	3	15	4	A	111	49.71866667	-66.99316667	4.79	33.83	105.45	171
117_1	2023-09-02	Pos	Pos	M	259	116	8	1.12	2	A	117	49.14233333	-66.81633333	6.19	34.49	56.14	252
117_3	2023-09-02	Pos	Neg	F	262	142	7	4.34	3	A	117	49.14233333	-66.81633333	6.19	34.49	56.14	252
117_4	2023-09-02	Pos	Neg	M	265	134	8	4.8	3	A	117	49.14233333	-66.81633333	6.19	34.49	56.14	252
117_5	2023-09-02	Pos	Neg	M	257	118	7	2.2	8	A	117	49.14233333	-66.81633333	6.19	34.49	56.14	252
118_3	2023-09-02	Neg	Neg	F	266	116	8	1.82	2	A	118	49.254	-66.36366667	6.02	34.4	63.13	243
118_4	2023-09-02	Pos	Neg	M	264	124	7	1.5	2	P	118	49.254	-66.36366667	6.02	34.4	63.13	243
118_5	2023-09-02	Pos	Neg	M	265	132	8	8.3	4	A	118	49.254	-66.36366667	6.02	34.4	63.13	243

Sample	Date	ENV	Ichty	Sex	Length	Weight	Age	Gon.weight	Maturity	Spawn Group	Station	Latitude	Longitude	Temperature	Salinity	Oxygene	Depth
**S17-1**	2024-09-27	Pos	Pos	NA	NA	NA	NA	NA	NA	NA	M3I	47.81666667	-65.21666667	NA	NA	NA	25
**S17-2**	2024-09-27	Neg	Neg	NA	NA	NA	NA	NA	NA	NA	M3I	47.81666667	-65.21666667	NA	NA	NA	25
**S17-3**	2024-09-27	Pos	Neg	NA	NA	NA	NA	NA	NA	NA	M3I	47.81666667	-65.21666667	NA	NA	NA	25
**S17-4**	2024-09-27	Pos	Neg	NA	NA	NA	NA	NA	NA	NA	M3I	47.81666667	-65.21666667	NA	NA	NA	25
**S17-5**	2024-09-27	Pos	Neg	NA	NA	NA	NA	NA	NA	NA	M3I	47.81666667	-65.21666667	NA	NA	NA	25
**S17-6**	2024-09-27	Pos	Neg	NA	NA	NA	NA	NA	NA	NA	M3I	47.81666667	-65.21666667	NA	NA	NA	25
**S17-8**	2024-09-27	Pos	Neg	NA	NA	NA	NA	NA	NA	NA	M3I	47.81666667	-65.21666667	NA	NA	NA	25
**S17-9**	2024-09-27	Pos	Neg	NA	NA	NA	NA	NA	NA	NA	M3I	47.81666667	-65.21666667	NA	NA	NA	25
**S17-10**	2024-09-27	Pos	Neg	NA	NA	NA	NA	NA	NA	NA	M3I	47.81666667	-65.21666667	NA	NA	NA	25
**S18-1**	2024-09-28	Pos	Neg	NA	NA	NA	NA	NA	NA	NA	M7I	47.85	-65.06666667	NA	NA	NA	22
**S18-2**	2024-09-28	Pos	Neg	NA	NA	NA	NA	NA	NA	NA	M7I	47.85	-65.06666667	NA	NA	NA	22
**S18-3**	2024-09-28	Pos	Neg	NA	NA	NA	NA	NA	NA	NA	M7I	47.85	-65.06666667	NA	NA	NA	22
**S18-4**	2024-09-28	Pos	Neg	NA	NA	NA	NA	NA	NA	NA	M7I	47.85	-65.06666667	NA	NA	NA	22
**S18-5**	2024-09-28	Pos	Pos	NA	NA	NA	NA	NA	NA	NA	M7I	47.85	-65.06666667	NA	NA	NA	22
**S18-6**	2024-09-28	Pos	Neg	NA	NA	NA	NA	NA	NA	NA	M7I	47.85	-65.06666667	NA	NA	NA	22
**S18-7**	2024-09-28	Pos	Neg	NA	NA	NA	NA	NA	NA	NA	M7I	47.85	-65.06666667	NA	NA	NA	22
**S18-8**	2024-09-28	Neg	Neg	NA	NA	NA	NA	NA	NA	NA	M7I	47.85	-65.06666667	NA	NA	NA	22
**S18-9**	2024-09-28	Neg	Neg	NA	NA	NA	NA	NA	NA	NA	M7I	47.85	-65.06666667	NA	NA	NA	22
**S25-1**	2024-09-30	Neg	Neg	NA	230	NA	NA	NA	NA	NA	S8O	48.11666667	-64.98333333	NA	NA	NA	20
**S25-2**	2024-09-30	Neg	Neg	NA	250	NA	NA	NA	NA	NA	S8O	48.11666667	-64.98333333	NA	NA	NA	20
**S25-3**	2024-09-30	Neg	Neg	NA	300	NA	NA	NA	NA	NA	S8O	48.11666667	-64.98333333	NA	NA	NA	20
**S25-4**	2024-09-30	Neg	Neg	NA	290	NA	NA	NA	NA	NA	S8O	48.11666667	-64.98333333	NA	NA	NA	20
**S25-5**	2024-09-30	Neg	Neg	NA	320	NA	NA	NA	NA	NA	S8O	48.11666667	-64.98333333	NA	NA	NA	20
**S25-6**	2024-09-30	Neg	Neg	NA	215	NA	NA	NA	NA	NA	S8O	48.11666667	-64.98333333	NA	NA	NA	20
**S25-7**	2024-09-30	Neg	Neg	NA	220	NA	NA	NA	NA	NA	S8O	48.11666667	-64.98333333	NA	NA	NA	20
**S25-8**	2024-09-30	Neg	Neg	NA	280	NA	NA	NA	NA	NA	S8O	48.11666667	-64.98333333	NA	NA	NA	20
**S25-9**	2024-09-30	Neg	Neg	NA	300	NA	NA	NA	NA	NA	S8O	48.11666667	-64.98333333	NA	NA	NA	20
**S25-10**	2024-09-30	Neg	Neg	NA	280	NA	NA	NA	NA	NA	S8O	48.11666667	-64.98333333	NA	NA	NA	20
**S27-1**	2024-09-30	Pos	Neg	NA	270	NA	NA	NA	NA	NA	S5I	48.046333	-65.147	NA	NA	NA	18
**S27-2**	2024-09-30	Pos	Neg	NA	210	NA	NA	NA	NA	NA	S5I	48.046333	-65.147	NA	NA	NA	18
**S27-3**	2024-09-30	Neg	Neg	NA	290	NA	NA	NA	NA	NA	S5I	48.046333	-65.147	NA	NA	NA	18
**S27-4**	2024-09-30	Neg	Neg	NA	270	NA	NA	NA	NA	NA	S5I	48.046333	-65.147	NA	NA	NA	18
**S27-5**	2024-09-30	Neg	Neg	NA	215	NA	NA	NA	NA	NA	S5I	48.046333	-65.147	NA	NA	NA	18
**S27-6**	2024-09-30	Neg	Neg	NA	205	NA	NA	NA	NA	NA	S5I	48.046333	-65.147	NA	NA	NA	18
**S27-7**	2024-09-30	Neg	Neg	NA	225	NA	NA	NA	NA	NA	S5I	48.046333	-65.147	NA	NA	NA	18
**S27-8**	2024-09-30	Neg	Neg	NA	255	NA	NA	NA	NA	NA	S5I	48.046333	-65.147	NA	NA	NA	18
**S27-9**	2024-09-30	Neg	Neg	NA	220	NA	NA	NA	NA	NA	S5I	48.046333	-65.147	NA	NA	NA	18
**S27-10**	2024-09-30	Neg	Neg	NA	215	NA	NA	NA	NA	NA	S5I	48.046333	-65.147	NA	NA	NA	18
**S30-1**	2024-10-04	Neg	Neg	NA	295	NA	NA	NA	NA	NA	NC4O	47.972833	-65.508167	NA	NA	NA	22
**S30-2**	2024-10-04	Neg	Neg	NA	220	NA	NA	NA	NA	NA	NC4O	47.972833	-65.508167	NA	NA	NA	22
**S30-3**	2024-10-04	Neg	Neg	NA	215	NA	NA	NA	NA	NA	NC4O	47.972833	-65.508167	NA	NA	NA	22
**S30-4**	2024-10-04	Neg	Neg	NA	255	NA	NA	NA	NA	NA	NC4O	47.972833	-65.508167	NA	NA	NA	22
**S30-5**	2024-10-04	Neg	Neg	NA	210	NA	NA	NA	NA	NA	NC4O	47.972833	-65.508167	NA	NA	NA	22
**S30-6**	2024-10-04	Neg	Neg	NA	250	NA	NA	NA	NA	NA	NC4O	47.972833	-65.508167	NA	NA	NA	22
**S30-7**	2024-10-04	Neg	Neg	NA	225	NA	NA	NA	NA	NA	NC4O	47.972833	-65.508167	NA	NA	NA	22
